# Critique of omnipolar mapping claims in superior vena cava isolation: A call for standardization

**DOI:** 10.1002/joa3.70020

**Published:** 2025-02-07

**Authors:** Mirza Muhammad Hadeed Khawar, Muneeb Khawar, Javed Iqbal, Abdul Qadeer

**Affiliations:** ^1^ Department of Cardiology Services Institute of Medical Sciences Lahore Lahore Pakistan; ^2^ Department of Cardiology King Edward Medical University Lahore Lahore Pakistan; ^3^ Nursing Department Hamad Medical Corporation Doha Qatar; ^4^ Department of Internal Medicine Mayo Clinic Hospital Phoenix Arizona USA

Dear Editor,

We write to address critical issues in the main findings of the study, “*Novel Omnipolar Mapping Technology for Effective Superior Vena Cava Isolation: A Randomized Clinical Trial*” by Oguri et al.^1^ While the study aims to demonstrate the superiority of omnipolar mapping technology (OT) over conventional methods (CM) in superior vena cava (SVC) isolation, the results are undermined by several methodological inconsistencies, questionable conclusions, and significant gaps in transparency. These issues call into question the study's overall validity and its implications for clinical practice.

The authors report that OT requires fewer radiofrequency (RF) applications (13.6 ± 6.0 vs 19.8 ± 10.9) and shorter procedure times (9.6 ± 6.8 min vs 14.3 ± 6.8 min) compared to CM. Although these differences are statistically significant, their clinical relevance is dubious because of the absence of clearly defined procedural criteria. The reported RF applications for OT are notably higher than the averages documented in previous SVC isolation studies,^2^ which typically highlight more efficient procedural workflows. This discrepancy raises concerns about whether OT genuinely represents an advancement or merely reflects methodological differences.

Moreover, the lack of transparency regarding operator variability—such as differences in experience levels and techniques—further complicates interpretation. Operator‐dependent factors are known to substantially impact RF application times and procedural outcomes; yet the study does not adequately address these influences. Without controlling for such variability, the reported superiority of OT remains speculative and difficult to generalize.

The authors claim that OT identified the SN location in three out of 25 patients where bipolar mapping failed. While this assertion suggests a potential advantage of OT, the study does not provide robust quantitative evidence to support the claim. For example, no clear validation framework for comparing the accuracy of OT versus bipolar mapping was included, nor was there external confirmation of the identified SN locations. Without such standardization, conclusions regarding OT's accuracy are speculative at best.

In a related analysis, the study describes the performance of an 8‐spline catheter, reporting higher point density (59 ± 10 vs 18 ± 4 electrograms/cm^2^; *p* < .01) and faster point acquisition rates (1332 ± 208 vs 308 ± 69 electrograms/min; *p* < .01) compared to the 5‐spline catheter during sustained atrial tachycardia mapping.^3^ However, these results, while statistically impressive, fail to demonstrate clinical relevance in the context of SN localization. The lack of consistent criteria for measuring and validating mapping accuracy undermines the credibility of these findings.

The study defines RA‐SVC conduction blocks using a color spectrum variability method, a departure from established electrophysiological criteria. This unconventional approach raises significant concerns about the validity of the reported findings. As highlighted in Roney et al.,^4^ standardized definitions and methodologies are crucial for interpreting complex electrophysiological phenomena, such as conduction blocks. The variability in methodology observed in the current study casts doubt on whether the identified block lines truly represent conduction blocks or are artifacts of the chosen mapping system.

Furthermore, the study's use of proprietary technology and software introduces another layer of complexity. Without external validation or reproducibility using other mapping platforms, the findings cannot be generalized to broader clinical settings. This is a critical limitation that undermines the study's ability to inform clinical decision‐making.

In conclusion, the main findings of the study by Oguri et al., including claims about RF applications, SN localization, and procedural safety, are weakened by significant methodological flaws and unsubstantiated assumptions. The absence of standardized criteria for key endpoints, combined with limited transparency regarding operator variability and procedural definitions, diminishes the study's overall impact and raises concerns about the purported advantages of omnipolar mapping technology.

A reevaluation of the data using standardized protocols, rigorous validation methods, and transparent reporting is essential to substantiate OT's claimed benefits. Until such steps are taken, it remains premature to advocate for the routine use of OT in SVC isolation based on the current evidence.

Sincerely,

Mirza Muhammad Hadeed Khawar.

## FUNDING INFORMATION

No funding was received for the preparation or submission of this letter.

## CONFLICT OF INTEREST STATEMENT

Authors declare no conflict of interests for this article.

## ETHICS APPROVAL STATEMENT

As this is a commentary on a published study and no new data were collected or analyzed, ethics approval was not required.

## Data Availability

This letter to the editor is a critique of previously published data by Oguri et al.^1^ All data referenced in this letter are publicly available in the original publication and cited sources. No new data were generated or analyzed in the preparation of this letter.
